# Detection of femtosecond spin voltage pulses in a thin iron film

**DOI:** 10.1063/4.0000037

**Published:** 2020-11-05

**Authors:** K. Bühlmann, G. Saerens, A. Vaterlaus, Y. Acremann

**Affiliations:** Laboratory for Solid State Physics, ETH Zurich, 8093 Zurich, Switzerland

## Abstract

We present experimental evidence of a spin voltage—a difference between the chemical potentials of the two spin directions—in a thin iron film based on spin- and time-resolved photoemission spectroscopy. This voltage is the driving force for a spin current during the ultrafast demagnetization of the sample. The observed magnitude is on the order of 50 mV, a value that is quite consistent with predictions based on particle conservation and persists for approximately 100 fs.

## INTRODUCTION

In conventional electronics, we use the charge of an electron to transport and process information. In contrast, spintronic devices use the electron spin as an additional degree of freedom. This has the consequence that the current density consists of two components: a current density of spins along and against the quantization direction. This model has already been proposed in 1936 by Mott^1^ to explain the temperature-dependent conductivity of ferromagnetic metals. The full two-current model includes the spin-dependent transport properties as well as spin flips.^2–4^ Only with the development of clean, ultrathin ferromagnet—non-magnetic metal heterostructures—it has become possible to utilize spin-transport effects in magnetic giant magneto-resistance (GMR) sensors.^5–7^ This effect is routinely applied in modern hard disk read heads. With the development of laterally nanostructured spin valves, it has been discovered that the magnetization of a ferromagnet can not only be detected by spin-dependent transport experiments but can even be manipulated by strong spin currents.^8–11^ This mechanism, in combination with the magneto-resistance effect, is the key to developing magnetic random access memory devices.^12^

To date, most experiments on spintronics have been performed at low frequencies. However, the ultrafast demagnetization effect^13^ led to a source of femtosecond spin current pulses: If a ferromagnet becomes demagnetized by a femtosecond laser pulse, part of the spin angular momentum is transported from the ferromagnet to the substrate.^14–16^ This generated femtosecond spin current pulse extends the field of spintronics into the ultrafast time domain.

There is an analogy for the voltage, charge, and current in spintronics: The spin voltage (which is the driving force for a spin current) is given by the difference between the spin-split electrochemical potentials as $V_{s}=\frac{\mu_{↑}-\mu_{↓}}{e}$, where *e* is the elemental charge. The spin current density is the difference between the minority and majority current densities: $j_{s}=j_{↑}-j_{↓}$. These quantities are difficult to measure on the femtosecond time scale. Kampfrath *et al.*^17^ demonstrated that the spin current density can be detected on the sub-picosecond time scale by THz spectroscopy: If a spin current is injected into a gold layer, the spin-Hall effect causes a charge current pulse, which emits THz radiation. This effect leads to an efficient method for the generation of THz radiation.^18^ The resistivity for spin transport is also detectable by THz spectroscopy.^19^ However, the spin voltage, which acts as a driving force for the spin current^4^ and spin flips,^20^ is difficult to measure. In quasi-DC transport experiments, the spin voltage can be observed in nonlocal spin valves.^21^ Here, we demonstrate the observation of femtosecond spin voltage pulses by spin- and time-resolved photoelectron spectroscopy on an iron film.

## EXPERIMENTAL SETUP

An iron film with a thickness of approximately 20 monolayers was grown *in situ* on a single-crystalline tungsten [111] substrate. Epitaxial growth led to a magnetic anisotropy parallel to the $\left[1\underline{1}0\right]$ direction of the substrate. More specific properties of the sample together with a detailed description of the preparation process can be found in Ref. 22.

**FIG. 1.**
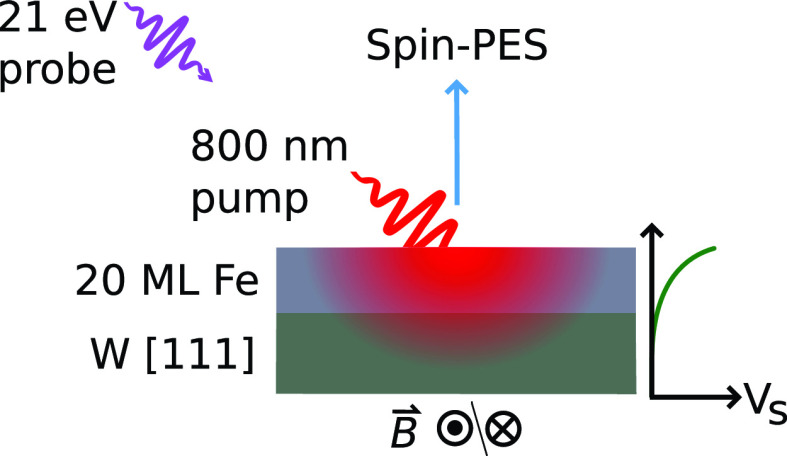
The sample consists of an iron film on W(111). Its magnetization is set by magnetic field pulses of 100 Oe peak amplitude and 12 μs length. The measurement is performed in remanence. The ferromagnet is excited by an 800 nm pump pulse (incident fluence: $5.6\;{\text{mJ}/\text{cm}}^{2}$). The pump pulse induces the formation of a temperature gradient in the Fe film, which generates a spin voltage gradient *V_s_*. The spin-split chemical potentials at the surface are detected by spin-resolved photoelectron spectroscopy (spin-PES). The electrons are emitted into the photoelectron spectrometer by a 21 eV photon pulse originating from a higher harmonic generation source.

Experiments were performed in the pump probe scheme based on a 10 kHz titanium sapphire amplifier laser system (Legend Elite, Coherent) with a center wavelength of 800 nm and a pulse duration of 20 fs. For excitation, the fundamental beam was used with an incident fluence of approximately $5.6\;\;{\text{mJ}/\text{cm}}^{2}$. The probe beam was frequency upconverted to 21 eV for photoemission by high harmonic generation (HHG). Emitted electrons were detected in a spin-, angle-, and energy-resolved setup, whereas spin resolution was provided by spin-polarized low-energy electron diffraction on an iridium crystal. A schematic of the sample and the setup is shown in Fig. 1. The experimental setup is described in detail in Refs. 23 and 24.

The photoelectron spectra were recorded for the magnetization in two directions $M_{↑},\;M_{↓}$. The magnetization of the sample was reversed every 10 s by a core-less field coil driven by a pulse generator.^25^ The magnetic field was 100 Oe with a pulse length of 12 μs. In order to avoid deflection of the photoelectrons by magnetic fields, the measurement was performed in remanence.

## PROCEDURE

**FIG. 2.**
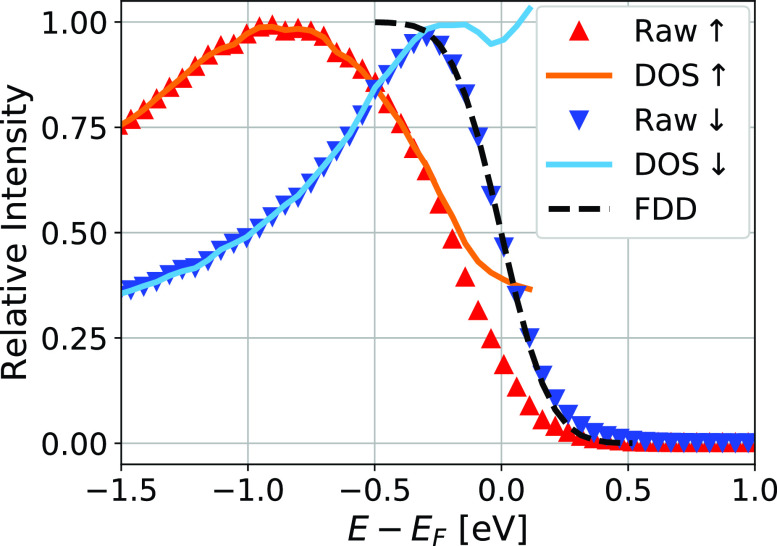
Reconstruction of unperturbed density of states from the raw spectra: The photoemission spectra (normalized such that the maximum value is 1) for majority (Raw ↑) and minority electrons (Raw ↓) are divided by the corresponding room-temperature Fermi–Dirac function convoluted with the point spread function given by the energy resolution. This leads to the density of states at room temperature (DOS ↑,↓). The black curve shows the Fermi–Dirac distribution convoluted with the point-spread function modeling the energy resolution.

To determine the spin-dependent chemical potentials $\mu_{↑}$ and $\mu_{↓}$, we need to determine the exact shape of the Fermi edge. For this, we need to know the density of states (DOS) within the acceptance window of our spectrometer. Once this is known, the time-dependent spectra can be divided by the DOS, resulting in the Fermi–Dirac function. Figure 2 shows the raw spectra of the electron gas before excitation by the pump laser. In order to determine the ground state DOS for each spin direction, these spectra are divided by *f_c_*, the convolution of the Fermi–Dirac function with the point-spread function modeling the energy resolution of our setup,

$$f_{c}=f(E,\mu ,T)*\frac{1}{\sqrt{2\pi }\sigma }e^{\frac{-E^{2}}{2\sigma^{2}}}.$$ (1)

Here, *E* is the energy relative to *E_f_*, *f* is the Fermi–Dirac function, *μ* is the chemical potential, and *σ* is the energy resolution of the setup. * denotes the convolution.

We restricted this analysis to $E-E_{f}<0.1\;\text{eV}$ because at higher energies, the number of electrons becomes too small for a robust determination of the DOS.

In principle, one could determine the Fermi edge by dividing the spin-resolved spectra by the ground state DOS. However, from Refs. 23 and 26–28, it is known that the spin-split DOS is altered once the sample is excited by the pump pulse: The spin-dependent DOS in the partially demagnetized state is a mixture of the original spin-split DOS terms. This is mathematically represented in Eq. (2), where ΔM(t) denotes the demagnetization and *M*_0_ is the ground state magnetization. This “band structure mirroring” is the result of the loss of long-range order and spatial averaging.^23,26,28^ The spin-resolved photoemission spectra $s_{↑,↓}$, therefore, have the shape

$$\begin{array}{c} s_{↑,↓}(E_{B},t)=f_{c}(T(t)),\\ \left({\text{DOS}}_{↑,↓}(E_{B})\left(1-\frac{\Delta M(t)}{2M_{0}}\right)+{\text{DOS}}_{↓,↑}(E_{B})\frac{\Delta M(t)}{2M_{0}}\right). \end{array}$$ (2)

**FIG. 3.**
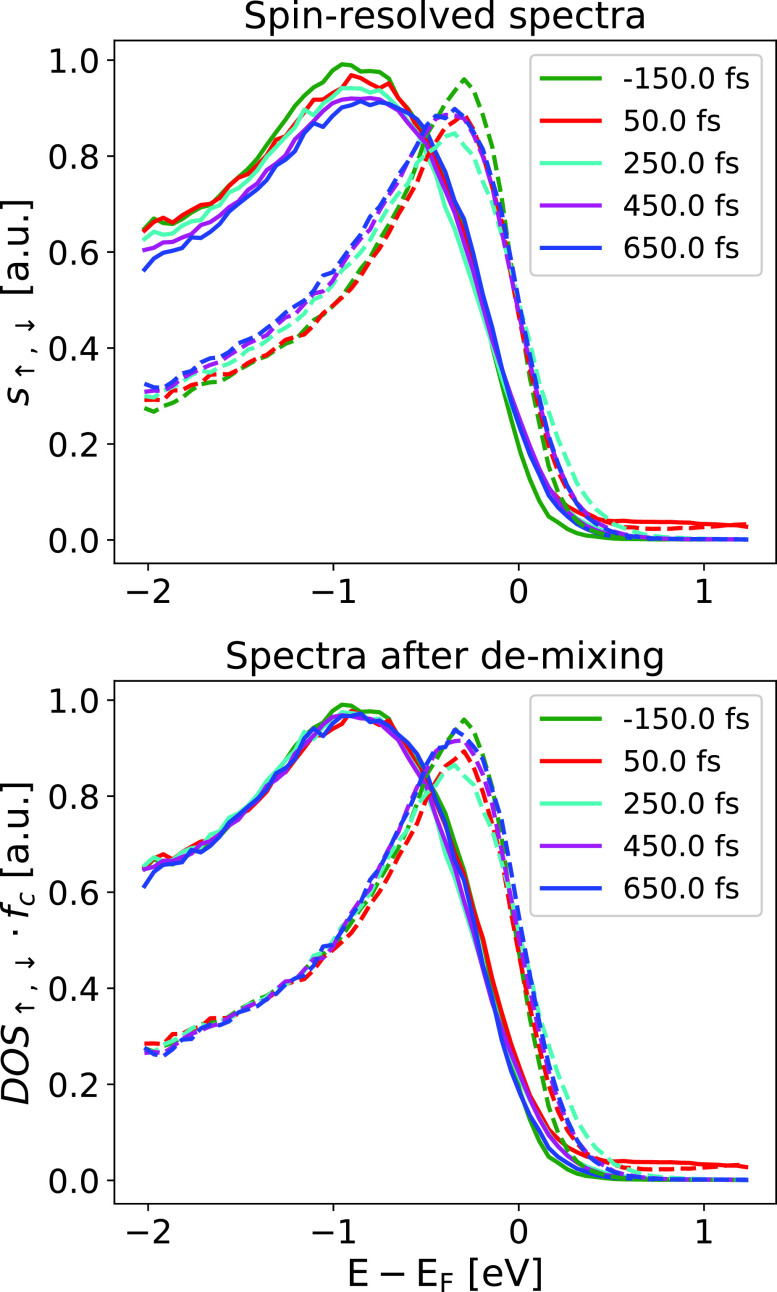
Demonstration of the reverting the band structure mirroring effect according to Eq. (2). The upper panel shows the spin-resolved spectra (majority electrons: solid lines; minority electrons: dashed lines). Even at 2 eV below for the Fermi edge, there are differences between the spectra due to the band structure mirroring effect.^28^ The lower panel shows the reconstruction of $\mathrm{DOS}f_{c}$ according to Eq. (2). Here, the spectra for $E-E_{f}<1\;\;\text{eV}$ are independent of the delay time.

The measured spectra $s_{↑,↓}(E_{B},t)$ are, therefore, given by the ground state DOS mixed proportionally by the demagnetization.^23^ The band structure mirroring process needs to be inverted in order to determine the product of the DOS with the Fermi-Dirac distribution. We demonstrate this in Fig. 3: on the upper panel, we show the measured spin-resolved spectra $s_{↑,↓}(E_{B},t)$ for selected time steps. Near the Fermi edge, one can see thermal broadening after the pump pulse. However, even at a binding energy of −2 eV, differences between the spectra are visible. As the Fermi–Dirac function is approximately 1 at this binding energy, the difference is a consequence of the band structure mirroring effect.^28^ On the lower panel, we show the result of calculating $DOS_{↑,↓}f_{c}$ by rearranging Eq. (2). We can see that $DOS_{↑,↓}f_{c}$ does not depend on time for $E-E_{f}<1\;\text{eV}$. The time dependence at close to the Fermi edge is caused by the temperature and chemical potential shifts.

Including all the findings discussed above, we can establish the following procedure to determine the Fermi edge as a function of the pump-probe delay.

First, we need to determine the ground-state DOS as well as the energy resolution. This can be done using the spectra for a pump-probe delay *t *<* *0,

(1) In a first step, we need to determine the energy resolution of our setup. We fit *f_c_* to the Fermi edge of spin-down data at a negative delay with fixed $\mu_{↓}=0\;\;\text{eV}$ and $T_{e}=300\;K$ by varying *σ*. The obtained energy resolution is σ=160  meV. We use the minority spectrum for this step since its DOS is approximately constant around *E_F_*. The fit is shown in Fig. 2 (labeled FDD).
(2) We divide the spin-resolved spectra by *f_c_* to obtain an experimental DOS for each spin direction (${\text{DOS}}_{↑}$ and ${\text{DOS}}_{↓}$), as displayed in Fig. 2.

Now, we know the spin-split ground-state DOS as well as our energy resolution. The next steps are needed to determine the chemical potentials $\mu_{↑,↓}(t)$ for the excited sample as a function of the pump-probe delay *t*.

**FIG. 4.**
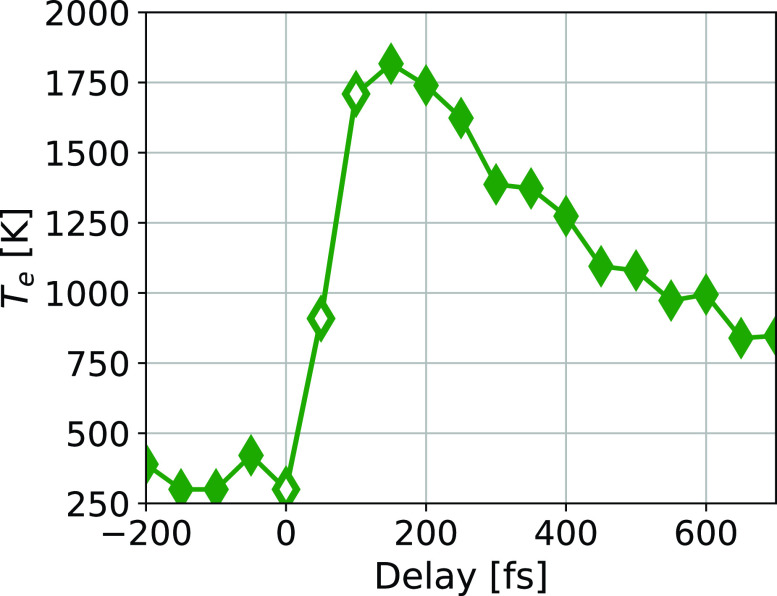
Evolution of the electron temperature. The three hollow markers indicate a nonthermal electron distribution; therefore, the temperature is not well defined, and the points only represent the output of the fitting process.

**FIG. 5.**
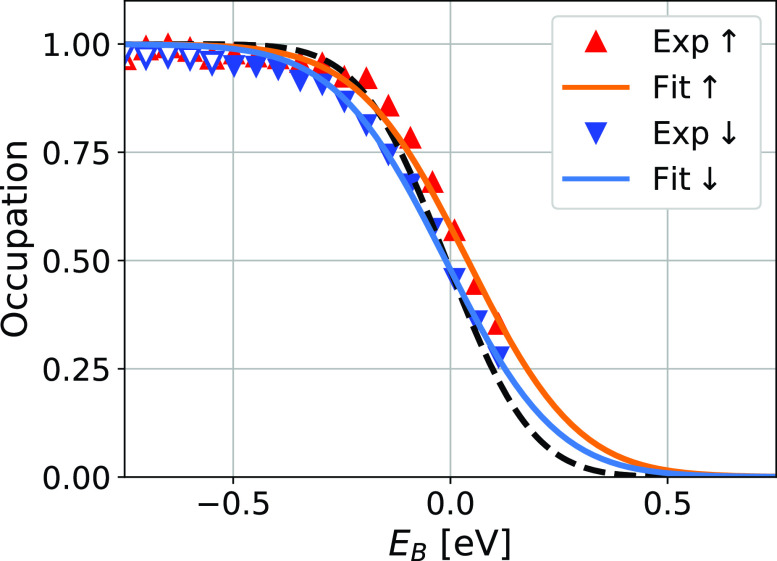
Experimental electron distributions and fits thereof at a delay of 50 fs. Only the filled markers were used for the fitting routine. The black curve shows the ground-state Fermi edge.

(3) First, we determine $DOS_{↑,↓}f_{c}$ from the measured spectra $s_{↑,↓}$ by the rearrangement of Eq. (2). These spectra are shown in Fig. 3, lower panel.
(4) We divide these spectra $DOS_{↑,↓}f_{c}$ by the ground-state $DOS_{↑,↓}$ (determined in step 2) to obtain the experimental electron occupation *f_exp_* for both spin directions.
(5) In order to get a robust value of the electron gas temperature, we fit *f_c_* to the spin-integrated spectra. The result is the electron temperature $T_{e}(t)$ (shown in Fig. 4). We justify this step by assuming that the electron gas for both spin directions has a common temperature due to the strong Coulomb coupling.
(6) The spin-dependent chemical potential can be determined by fitting *f_c_* to the measured electron occupation *f_exp_* for both spin directions. Note that we only have the chemical potentials $\mu_{↑,↓}$ left as free parameters: The electron gas temperature is determined in the last step. Figure 5 shows the fit of *f_exp_* for the pump-probe delay t=50 fs.

The difference between the chemical potentials of the two spin directions divided by the elemental charge *e* is the spin voltage. Its gradient along the surface normal is the driving force of the spin current,^16^

$$V_{S}(t)=\frac{\mu_{↑}(t)-\mu_{↓}(t)}{e}.$$ (3)

## RESULTS AND DISCUSSION

The evolution of the electron temperature is shown in Fig. 4. For the first hundred femtoseconds, the electron system did not thermalize; therefore, the temperature is not well defined during that time, as indicated by the hollow markers in the plot. We still showed these points, as they were used in the determination of the chemical potentials (see reasoning below). The increase in the electron temperature to a point well above the melting point of iron is not an issue, as the lattice will reach pseudo-equilibrium with the electrons only after approximately one picosecond. The maximum electron temperature was approximately 1800 K. This is consistent with the two-temperature model.^29^

**FIG. 6.**
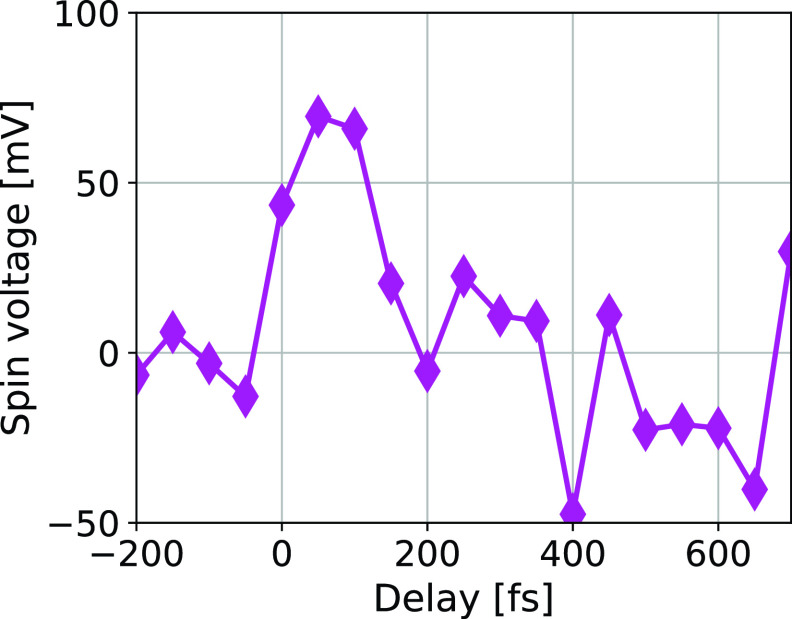
Evolution of the spin voltage as a function of time. The maximum voltage was reached at 50 fs after the pump pulse. Its magnitude is in line with the model from Ref. 16.

The spin voltage displayed in Fig. 6 increased to a value of ≈50  mV upon the incidence of the pump pulse. The signal persisted for ≈100 fs, which is on the same timescale as the fastest part of the demagnetization in iron.^23^ In Ref. 16, a spin voltage of 30 mV for an electron temperature of $T_{e}=2000\;K$ at the sample's surface was calculated. This is in line with our observation.

As the occurrence of the spin voltage coincides with the nonthermal electron distribution, we needed to confirm that the former is not just an artifact of the latter. At 0 fs and 100 fs, the electrons followed a Fermi-Dirac distribution quite closely. However, at 50 fs, the system was far from thermalization, and the spectral intensity of nonthermal electrons above the Fermi energy was approximately 10% of the spectral intensity of those found below it. In Fig. 5, we show the experimental electron distributions for both spin directions at this delay together with their corresponding fits. Only the points represented by filled markers were used for the fitting routine. The obtained spin voltage is directly visible in the data and, therefore, not just caused by an artifact of the nonthermal electrons.

## CONCLUSIONS

The generation of femtosecond spin currents extends the field of spintronics to the ultrafast time domain. In spintronics, the concept of spin voltage plays the same role as the voltage in charge-based electronics: it is the driving force for the (spin-) current. Here, we experimentally observe the generation of a spin voltage pulse by spin- and time-resolved photoelectron spectroscopy. We observe that the spin voltage pulse lasts for only ≈100  fs and that its magnitude is in line with theoretical predictions.^16^ The spin voltage gradient is the driving force for a pure spin current.

## SUPPLEMENTARY MATERIAL

See the supplementary material for the spectra and the analysis software.

## Data Availability

The data that support the findings of this study are available within this article and its supplementary material.
